# Associations of health-related fitness and physical activity with chemotherapy outcomes in breast cancer

**DOI:** 10.1038/s41416-026-03384-3

**Published:** 2026-03-27

**Authors:** Renée L. Kokts-Porietis, Andria R. Morielli, Lin Yang, Charles E. Matthews, Sasha Lupichuk, Gloria Roldan-Urgoiti, Margaret L. McNeely, S. Nicole Culos‑Reed, Jeff K. Vallance, Leanne Dickau, Kerry S. Courneya, Christine M. Friedenreich

**Affiliations:** 1 Department of Cancer Epidemiology and Prevention Research, Cancer Care Alberta, Calgary, AB Canada; 2 Cancer Prevention and Screening Innovation, Health Intelligence, Evidence and Improvement, Planning and Quality, Primary Care Alberta, Calgary, AB Canada; 3 Department of Oncology, Cumming School of Medicine, University of Calgary, Calgary, AB Canada; 4 Department of Community Health Sciences, Cumming School of Medicine, University of Calgary, Calgary, AB Canada; 5 Division of Cancer Epidemiology and Genetics, US National Cancer Institute, Rockville, MD USA; 6 Faculty of Rehabilitation Medicine, University of Alberta, Edmonton, AB Canada; 7 Faculty of Kinesiology, University of Calgary, Calgary, AB Canada; 8 Faculty of Health Disciplines, Athabasca University, Athabasca, AB Canada; 9 Faculty of Kinesiology, Sport, and Recreation, College of Health Sciences, University of Alberta, Edmonton, AB Canada

**Keywords:** Breast cancer, Chemotherapy, Cancer epidemiology

## Abstract

**Background:**

Limited research exists on how modifiable lifestyle factors influence tolerability and response to chemotherapy. We investigated the associations between health-related fitness, physical activity, and sedentary behaviour after diagnosis with relative dose intensity (RDI) and pathologic complete response (pCR) among newly diagnosed breast cancer patients who received neoadjuvant or adjuvant chemotherapy.

**Methods:**

This analysis includes 890 participants of the Alberta Moving Beyond Breast Cancer prospective cohort study who received chemotherapy. We directly measured cardiorespiratory fitness, muscular strength and endurance, body composition, physical activity, sedentary behaviour shortly after diagnosis and abstracted RDI and pCR data from medical charts. We used logistic regression to measure the associations with RDI ( < 85%, ≥ 85%) and pCR (no, yes).

**Results:**

Of 890 participants, 726 (81.6%) achieved ≥85% RDI. We found negative linear associations between ≥ 85% RDI and greater body mass index (BMI), waist circumference, waist-to-hip ratio and fat mass percentage; and positive linear associations for greater lean body mass percentage and lean-to-fat mass ratio. We observed positive dose-response relationships between ≥ 85% RDI and relative VO_2peak_, upper body and lower body strength. Higher lean-to-fat mass ratio was positively associated with pCR, in contrast to negative associations for BMI and self-reported physical activity.

**Conclusion:**

Greater relative aerobic fitness, muscular strength, and healthy body composition were consistently associated with better chemotherapy tolerance whereas fewer associations were found with chemotherapy response.

## Background

Breast cancer is the most frequently diagnosed cancer and the leading cause of cancer death among women worldwide [1]. In curative-intent breast cancer treatment, chemotherapy remains the most common systemic modality. Unlike surgery, which is typically a one-time intervention, chemotherapy requires sustained administration over multiple cycles [2]. Hence, patient prognosis and overall survival benefits associated with chemotherapy depend on delivery of the planned dose and treatment schedule [2, 3].

Despite prognostic benefits, chemotherapy induces serious adverse events that result in dose reductions and delays. Relative dose intensity (RDI), the ratio of delivered to standard or planned chemotherapy dose (range 0–100%), accounting for delay, reductions, and discontinuations, is a key indicator of treatment tolerability and adherence [4]. RDI ≥85% is considered a relevant marker of successful chemotherapy delivery for optimal prognostic benefits [4]. In the neoadjuvant setting, pathologic complete response (pCR), defined as the absence of residual invasive cancer in the breast and all sampled regional lymph nodes post neoadjuvant chemotherapy, also serves as a strong prognostic and predictive indicator [5].

Several factors may influence failing to achieve ≥ 85% RDI, including chemotherapy regimens, age, body surface area, comorbidity burden, and febrile neutropenia [6–8]. Less is known about the role of modifiable lifestyle factors. Evidence to date suggests that physical activity (PA) [9] and health-related fitness (HRF) factors including cardiorespiratory fitness [10, 11], muscular strength [10, 11], and body composition [12] may impact treatment completion and pCR [13] in women with breast cancer. Although sedentary behaviour (SB) has been associated with survival outcomes after cancer, it has not been investigated in relation to RDI and pCR. To date [9, 12], findings remain inconsistent due to variability in outcome measurement [9], retrospective study designs [12], and heterogeneity of HRF measurements [12].

To address these knowledge gaps, we investigated how HRF, PA, and SB are associated with chemotherapy completion and response in a prospective cohort of breast cancer patients who received (neo)adjuvant chemotherapy. The primary objective was to examine the associations between aerobic fitness, muscular strength, muscular endurance, body composition, PA, and SB with chemotherapy RDI in breast cancer patients initiating (neo)adjuvant chemotherapy in the Alberta Moving Beyond Breast Cancer (AMBER) Cohort. An exploratory objective was to examine their associations with pCR in a neoadjuvant chemotherapy subgroup.

## Methods

### Participants and Setting

The AMBER Study is a prospective cohort of newly diagnosed breast cancer patients recruited between 2012 and 2019 in Alberta, Canada. Full cohort details have been previously described [14]. Briefly, women 18–80 years of age, diagnosed with primary stage I-IIIc breast cancer and able to complete the revised PA Readiness Questionnaire for Everyone were eligible to participate. Of the 1528 participants, 890 underwent neoadjuvant or adjuvant chemotherapy as part of their primary treatment and met the eligibility criteria for this analysis. We excluded those participants who did not undergo chemotherapy. We obtained ethics approval through the Health Research Ethics Board of Alberta: Cancer Committee (HREBA.CC-17-0576). All participants provided informed, written consent.

### Data Collection and Measurements

Among participants who received chemotherapy, we obtained baseline characteristics, PA, SB, and HRF measurements with a median of 82 [interquartile ranges (IQR) = 66–97] days after their initial cancer diagnosis. We aimed to complete these assessments prior to initiation of chemotherapy however, given individual participant circumstances, 275 (30.9%) assessments were done after adjuvant treatment had been initiated. At baseline, participants reported sociodemographic characteristics, average daily SB, family history of cancer, medical, reproductive, exogenous hormone, lifetime smoking, and alcohol use histories. Comorbidities were summarized using the Charlson Comorbidity Index [15]. Self-reported PA for the year before diagnosis of breast cancer was captured with the *Past Year Total PA Questionnaire* [16].

Trained exercise physiologists assessed participants’ cardiorespiratory fitness with a graded treadmill exercise test using the modified Bruce protocol to determine absolute and relative peak volume of oxygen consumed (VO_2peak_). Dual x-ray absorptiometry (DXA) measured participants’ body composition including fat percentage, lean mass percentage, and lean-to-fat mass ratio. They measured height and weight to calculate body mass index (BMI) and waist circumference and hip circumferences to generate waist-to-hip ratio (WHR). They assessed maximal upper and lower body strength in the seated position by a predicted one repetition maximum (1-RM) from an 8–10 repetition chest press (APEX Vertical Bench) and seated leg press (Atlantis C-403) maximum test, respectively. Muscular endurance was assessed as a multiple repetition maximum based on 50% of predicted 1-RM for chest press and 70% of predicted 1-RM for leg press and calculated as the total weight by number of repetitions lifted.

For the seven days following the HRF assessment, bouts of MVPA lasting ≥10 min and total energy expenditure (MET-h/d) were estimated via the waist-worn ActiGraph GT3X + ^®^ (ActiGraph, LLC, Pensacola, Florida) device and computed with the R Sojourn package (version 1.1.0, Soj3x) [17]. During this time, total sedentary time (primary lying+sedentary time) and average daily step counts were measured using the thigh-worn activPAL^®^ device (PAL Technologies, Glasgow, Scotland) and algorithms (PAL Software version 8). Participants wore the devices during their waking hours of the day with *n* = 197 (22%) also wearing the devices while sleeping. For both devices, we excluded days with < 10 h of wear time while out of bed resulting in the exclusion of four participants’ activPAL^®^ data.

### Chemotherapy Outcomes

A trained staff member abstracted the dose of chemotherapy agents in each treatment cycle, expressed as a percent of the intended dose given, as well as the timing of chemotherapy cycles, from participants’ medical charts. We estimated the RDI of neoadjuvant or adjuvant chemotherapy, excluding any targeted therapies, based on the method of Longo et al [18]. RDI < 85% was considered clinically suboptimal [4, 8, 19]. For participants who underwent neoadjuvant chemotherapy, we assessed pCR (ypT0/Tis ypN0) using the American Joint Committee on Cancer 7th edition staging criteria per the NeoSTEEP (Standardized Definitions for Efficacy End Points) guidelines [5].

### Statistical Analysis

To categorize BMI and WHR, we used the World Health Organization standards (BMI: <24.9, 25–29.9, ≥ 30 kg/m^2^; WHR: < 0.85, ≥ 0.85) [20]. Waist circumference was dichotomized based on the Canadian clinical threshold of 88 cm [20]. We used tertiles to group the remaining exposure variables. For cardiorespiratory fitness, muscular strength and endurance exposures, we included the inability to complete the assessment due to medical reasons in the assessment.

We summarized participant characteristics (overall and by neoadjuvant vs. adjuvant chemotherapy) using medians and IQR or frequencies and percentages. Logistic regression estimated multivariable-adjusted odds ratios (OR) and 95% confidence intervals (95% CI) for HRF, PA and SB tertiles (T1-T3) with RDI ( < 85%, ≥ 85%) and pCR (yes, no) outcomes. We evaluated linear trends using finer units for each HRF variable in multivariable logistic regressions. We selected covariates a priori including: age at diagnosis (years), cancer stage (I, II, III), cancer grade (I, II, III), study location (Calgary, Edmonton), breast cancer subtype (triple negative, HR + /HER2-, HR-/HER2 + , HR + /HER2 + ) and Charlson Comorbidity Index based on biological plausibility. We adjusted for RDI in the pCR assessment. There was insufficient statistical evidence that marital status, parity, income, highest education attained, menopausal status, ever hormone therapy use, first degree female history of cancer, alcohol consumption, smoking pack-years, inclusion of targeted therapy, chemotherapy type or days of chemotherapy in relation to HRF assessment confounded the associations of interest. We conducted sensitivity analyses by restricting the study sample to those who had not started chemotherapy (*N* = 615) for both RDI (eTables 1, 2), pCR (eTable 3), and those not receiving chemotherapy (*N* = 839) as part of a randomized controlled trial (eTable 4).

To maintain sample size, we addressed missing data on covariates via multivariate imputations through chained equations, which included all correlated covariates in regression models [21]. Statistical tests were two-sided, and a *P*-value < 0.05 was considered statistically significant. We conducted all analyses with STATA 18 (StataCorp LLC, College Station, TX).

## Results

Of the 890 participants treated with chemotherapy (taxane-based: 53.9%, anthracycline/taxane-based: 44.9%), 726 (81.6%) achieved ≥ 85% RDI (Table 1). Of the 117 participants who received neoadjuvant chemotherapy, 34 (29.1%) had a pCR. Participants’ median age was 53.0 years (IQR = 45.2–60.2), 83.9% identified as white, 75.4% lived as married/common-law and 78.1% had >high school education (Table 1). Most participants had stage II (59.6%), grade 3 (63.4%) and HR + /HER2- (62.1%) breast cancer. Participants who achieved ≥ 85% RDI had greater relative and absolute VO_2peak_, upper and lower body strength and upper body endurance (Table 2).

**Table 1:** Characteristics of Participants by Neoadjuvant and Adjuvant Chemotherapy Status in Alberta Moving Beyond Breast Cancer (AMBER) study (*n* = 890).

Characteristics	All Median (IQR) / N (%)	Neoadjuvant Median (IQR) / *N* (%)	Adjuvant Median (IQR) / *N* (%)	*P*-value
Participants	890	117	773	
Age	53.01 (45.2–60.2)	49.56 (41.4–58.4)	53.44 (46.0–60.8)	< 0.01
Population Group				
White	747 (83.9%)	98 (83.8%)	649 (84.0%)	0.96
Other	143 (16.1%)	19 (16.2%)	124 (16.0%)	
Location				
Calgary	485 (54.5%)	49 (41.9%)	436 (56.4%)	< 0.01
Edmonton	405 (45.5%)	68 (58.1%)	337 (43.6%)	
Education				
High school or less	180 (20.8%)	17 (14.9%)	163 (21.7%)	0.28
College or trade school	274 (31.7%)	36 (31.6%)	238 (31.7%)	
University undergraduate degree	240 (27.8%)	33 (28.9%)	207 (27.6%)	
University graduate degree	170 (19.7%)	28 (24.6%)	142 (18.9%)	
Marital status				
Married or common-law	670 (75.3%)	87 (74.4%)	583 (75.4%)	0.80
Divorced, separated, widowed, single or never married	220 (24.7%)	30 (25.6%)	190 (24.6%)	
Income				0.93
<$50,000	107 (13.2%)	14 (13.0%)	93 (13.3%)	
$50,000-100,000	252 (31.2%)	32 (29.6%)	220 (31.4%)	
$100,000-150,000	189 (23.4%)	28 (25.9%)	161 (23.0%)	
>$150,000	260 (32.2%)	34 (31.5%)	226 (32.3%)	
Menopausal status				
Pre-menopausal	299 (33.6%)	52 (44.4%)	247 (32.0%)	< 0.01
Peri-/ Post-menopausal	591 (66.4%)	65 (55.6%)	526 (68.0%)	
Exogenous hormone use				
No	706 (79.3%)	88 (75.2%)	618 (79.9%)	0.24
Yes	184 (20.7%)	29 (24.8%)	155 (20.1%)	
Parity				
0	188 (21.1%)	29 (24.8%)	159 (20.6%)	0.55
1	104 (11.7%)	12 (10.3%)	92 (11.9%)	
≥2	598 (67.2%)	76 (65.0%)	522 (67.5%)	
Smoking status				< 0.01
Never	509 (57.2%)	89 (76.1%)	420 (54.3%)	
Former	298 (33.5%)	24 (20.5%)	274 (35.4%)	
Current	83 (9.3%)	4 (3.4%)	79 (10.2%)	
Alcohol status				
Lifetime abstainer	48 (5.4%)	6 (5.1%)	42 (5.5%)	0.83
Former	22 (2.5%)	2 (1.7%)	20 (2.6%)	
Current	815 (92.1%)	109 (93.2%)	706 (91.9%)	
Female family history of cancer				
No	631 (71.8%)	92 (79.3%)	539 (70.6%)	0.05
Yes	248 (28.2%)	24 (20.7%)	224 (29.4%)	
Charlson comorbidity score	0.5 (0.0–1.0)	0.3 (0.0–1.0)	0.5 (0.0–1.3)	< 0.01
Breast cancer disease stage				
I	234 (26.3%)	4 (3.4%)	230 (29.8%)	< 0.01
II	530 (59.6%)	76 (65.0%)	454 (58.7%)	
III	126 (14.2%)	37 (31.6%)	89 (11.5%)	
Breast cancer tumor grade				
1	37 (4.2%)	4 (3.4%)	33 (4.3%)	0.81
2	289 (32.5%)	36 (30.8%)	253 (32.7%)	
3	564 (63.4%)	77 (65.8%)	487 (63.0%)	
Breast cancer subtype				
triple negative	109 (12.2%)	21 (17.9%)	88 (11.4%)	< 0.01
HR + /HER2-	553 (62.1%)	54 (46.2%)	499 (64.6%)	
HR-/HER2+	47 (5.3%)	9 (7.7%)	38 (4.9%)	
HR + /HER2+	181 (20.3%)	33 (28.2%)	148 (19.1%)	
Chemotherapy regimen type				
Taxane-based	480 (53.9%)	30 (25.6%)	450 (58.2%)	< 0.01
Anthracycline & Taxane-based	400 (44.9%)	87 (74.4%)	313 (40.5%)	
Other	10 (1.1%)	0 (0.0%)	10 (1.3%)	
HRF vs. chemotherapy timing (days)	3.0 (−10.0–11.0)	−11.0 (–17.0–2.0)	4.0 (−5.0–13.0)	< 0.01

*HRF* Health related fitness, *HER2* human epidermal growth factor receptor 2; *P*-values based on the Wilcoxon rank-sum or Chi-square tests; May not sum to total sample size because of missing values.

**Table 2:** Health Related Fitness, Body Composition, Physical Activity and Sedentary Behaviour Characteristics of Participants by Relative Dose Intensity Status in the Alberta Moving Beyond Breast Cancer (AMBER) study (n = 890).

Exposures	All Median (IQR)	RDI < 0.85% Median (IQR)	RDI ≥ 0.85% Median (IQR)	*P*-value
Participants	890	164	726	
**Aerobic fitness**				
Relative VO_2_ peak (ml/kg/min)	26.5 (22.9–30.6)	24.7 (21.6–28.8)	27.0 (23.2–30.8)	< 0.01
Absolute VO_2_ peak (L/min)	1.9 (1.7–2.2)	1.9 (1.6–2.1)	1.9 (1.7–2.2)	0.02
**Muscular strength**				
Upper body strength (kg)	35.7 (29.7–42.0)	33.0 (27.0–40.1)	36.0 (29.7–44.0)	< 0.01
Lower body strength (kg)	95.0 (77.0–118.9)	89.2 (71.0–107.0)	95.1 (77.3–118.9)	< 0.01
**Muscular endurance**				
Upper body endurance (kg)	460.0 (341.0–598.9)	436.2 (326.0–544.4)	475.0 (347.7–613.6)	< 0.01
Lower body endurance (kg)	1159.1 (795.0–1657.0)	1125.0 (795.0–1514.0)	1177.1 (784.1–1659.1)	0.49
**Body composition**				
Body mass index (kg/m^2^)	26.5 (23.2–30.2)	27.9 (24.4–31.4)	26.2 (23.0–29.9)	< 0.01
Waist circumference (cm)	91.6 (82.3–101.0)	94.4 (85.0–104.7)	91.0 (82.0–100.3)	< 0.01
Waist-to-hip ratio	0.9 (0.8–0.9)	0.9 (0.8–0.9)	0.9 (0.8–0.9)	0.02
Fat mass percentage (%)	43.4 (38.3–48.0)	45.6 (40.4–49.6)	43.0 (37.5–47.6)	< 0.01
Lean mass percentage (%)	53.4 (49.1–58.2)	51.3 (47.5–56.1)	53.8 (49.4–58.6)	< 0.01
Lean-to-fat mass ratio	1.2 (1.0–1.5)	1.1 (1.0–1.4)	1.3 (1.0–1.6)	< 0.01
**Physical Activity**				
Self-reported total physical activity (MET-h/wk/yr)	122.5 (85.9–172.7)	121.4 (85.8–163.2)	123.4 (86.0–176.0)	0.33
Self-reported recreational physical activity (MET-h/wk/yr)	20.3 (9.2–38.5)	18.6 (8.4–38.7)	21.1 (9.3–38.5)	0.42
Total energy expenditure (MET-h/d)	16.9 (15.1–19.0)	16.4 (14.6–18.9)	16.9 (15.3–19.0)	0.07
Measured 10+ minutes bouts of MVPA (h/d)	0.2 (0.0–0.4)	0.2 (0.0–0.5)	0.2 (0.0–0.4)	0.29
Average daily steps	6753.6 (5074.7–8906.7)	6399.0 (4649.8–8351.1)	6792.8 (5195.4–8952.0)	0.06
**Sedentary Behaviours**				
Self-reported average sitting time (h/d)	9.1 (6.9–11.2)	9.5 (7.1–11.7)	9.0 (6.8–11.1)	0.47
Total sedentary hours (h/d)	9.1 (7.9–10.2)	9.2 (7.9–10.3)	9.1 (7.9–10.1)	0.60

*MVPA* moderate-to-vigorous physical activity, *P*-values based on the Wilcoxon rank-sum.

In multivariable adjusted models, we observed positive linear associations between relative VO_2peak_ (OR_per 1 ml/kg/min_ = 1.06, 95% CI = 1.02–1.10), upper body strength (OR_per 10 kg_ = 1.26, 95% CI = 1.02–1.56) and lower body strength (OR_per 10 kg_ = 1.08, 95% CI = 1.01–1.14) with ≥ 85% RDI (Table 3). Participants with higher relative VO_2peak_ had greater odds of achieving ≥ 85% RDI (OR_T2 vs T1_ = 1.74, 95% CI = 1.09–2.80; OR_T3 vs T1_ = 2.17, 95% CI = 1.28–3.65). We found statistically significant associations between RDI and upper body strength for participants in the second vs. first tertile of upper body strength (OR_T2 vs T1_ = 1.67, 95% CI = 1.05–2.65) (Table 3).

**Table 3:** Logistic Regression for Health-Related Fitness, Body Composition, Physical Activity and Sedentary Behaviour with ≥85% Relative Dose Intensity in the Alberta Moving Beyond Breast Cancer (AMBER) study (*n* = 890).

	Events ≥ 85/No. Participants	OR (95% CI)
**Aerobic fitness**		
Relative VO_2_peak (ml/kg/min)		
Did not complete assessment	49/67	0.93 (0.49–1.77)
T1: < 24.2	178/237	1.00
T2: 24.2–28.9	199/235	1.74 (1.09–2.80)
T3: > 28.9	206/233	2.17 (1.28–3.65)
Ptrend		0.006
per 1 ml/kg/min	583/705	1.06 (1.02–1.10)
Absolute VO_2_ peak (L/min)		
Did not complete assessment	49/67	0.72 (0.37–1.38)
T1: < 1.76	192/239	1.00
T2: 1.76–2.06	186/231	0.94 (0.58–1.50)
T3: > 2.06	205/235	1.51 (0.88–2.60)
Ptrend		0.124
per 0.1 L/min	583/705	1.05 (0.99–1.12)
**Muscular Strength**		
Upper body strength (kg)		
Did not complete assessment	79/101	1.27 (0.71–2.28)
T1: < 32.1	192/254	1.00
T2: 32.1–40.1	219/257	1.67 (1.05–2.65)
T3: >40.1	206/239	1.57 (0.96–2.57)
Ptrend		0.036
per 10 kg	617/750	1.26 (1.02–1.56)
Lower body strength (kg)		
Did not complete assessment	40/55	0.92 (0.46–1.83)
T1: < 83.3	225/289	1.00
T2: 83.3–107.0	204/240	1.39 (0.88–2.20)
T3: >107.0	222/259	1.53 (0.96–2.43)
Ptrend		0.023
per 10 kg	651/788	1.08 (1.01–1.14)
**Muscular Endurance**		
Upper body endurance (total kg)		
Did not complete assessment	80/102	0.99 (0.55–1.81)
T1: < 384	202/250	1.00
T2: 384–552.3	190/246	0.77 (0.49–1.20)
T3: > 552.3	219/247	1.64 (0.98–2.74)
Ptrend		0.06
per 10 kg	611/743	1.01 (0.99–1.02)
Lower body endurance (total kg)		
Did not complete assessment	39/54	0.69 (0.34–1.42)
T1: < 922.7	217/259	1.00
T2: 922.7–1475.0	204/256	0.75 (0.48–1.19)
T3: >1475.0	216/257	1.07 (0.66–1.75)
Ptrend		0.28
per 10 kg	638/773	1.00 (0.99–1.00)
**Body Composition**		
Body mass index (kg/m^2^)		
≤24.9	303/349	1.00
25.0–29.9	246/306	0.68 (0.44–1.04)
≥30	177/235	0.55 (0.35–0.86)
Ptrend		0.009
per 1 kg/m^2^	726/890	0.96 (0.94–0.99)
Waist circumference (cm)		
<88.0	315/370	1.00
≥88.0	411/520	0.72 (0.50–1.04)
Ptrend		0.006
per 1 cm	726/890	0.98 (0.97–0.99)
Waist-to-hip ratio		
≤0.84	271/318	1.00
≥0.85	455/572	0.72 (0.49–1.07)
Ptrend		0.025
per 0.1	726/890	0.73 (0.56–0.95)
Fat mass percentage (%)		
T1: <40.1	256/290	1.00
T2: 40.1–46.4	238/290	0.67 (0.42–1.09)
T3: >46.4	217/289	0.47 (0.30–0.76)
Ptrend		0.001
per 1%	711/869	0.96 (0.93–0.98)
Lean body mass percentage (%)		
T1: < 50.6	217/290	1.00
T2: 50.6–56.3	240/290	1.50 (0.99–2.27)
T3: > 56.3	254/289	2.03 (1.28–3.22)
Ptrend		0.001
per 1%	711/869	1.05 (1.02–1.08)
Lean-to-fat mass ratio		
T1: < 50.2	217/290	1.00
T2: 50.2–56.5	240/290	1.51 (0.99–2.29)
T3: >56.5	254/289	2.06 (1.30–3.26)
Ptrend		0.001
per 0.1	711/869	1.09 (1.03–1.14)
**Physical Activity**		
Self-reported total non-sedentary physical activity (MET-h/wk/yr)		
T1: < 98.1	226/283	1.00
T2: 98.1–152.7	229/282	1.11 (0.72–1.70)
T3: > 152.7	237/282	1.25 (0.79–1.96)
Ptrend		0.88
per 10 MET-h/wk/yr	692/847	1.00 (0.97–1.03)
Self-reported recreational physical activity (MET-h/wk/yr)		
T1: < 12.7	229/283	1.00
T2: 12.7–31.5	231/282	1.09 (0.71–1.68)
T3: > 31.5	232/282	1.09 (0.70–1.68)
Ptrend		0.92
per 10 MET-h/wk/yr	692/847	1.00 (0.94–1.07)
Total energy expenditure (MET-h/d)		
T1: < 15.8	217/278	1.00
T2: 15.8–18.2	231/277	1.37 (0.88–2.11)
T3 > 18.2	229/277	1.17 (0.76–1.81)
Ptrend		0.32
per 1 MET-h/d	677/832	1.03 (0.97–1.09)
Measured 10+ minutes bouts of MVPA (h/d)		
T1: < 0.1	216/278	1.00
T2: 0.1–0.3	233/277	1.59 (1.02–2.48)
T3: > 0.3	228/277	1.31 (0.85–2.03)
Ptrend		0.91
per 1 h/d	677/832	1.03 (0.60–1.78)
Average daily steps (steps/d)		
T1: < 5506.0	217/275	1.00
T2: 5506.0–8061.5	227/274	1.10 (0.70–1.72)
T3: > 8061.5	231/274	1.24 (0.79–1.96)
Ptrend		0.26
per 1000 steps/d	675/823	1.04 (0.97–1.11)
**Sedentary Behaviours**		
Self-reported average sitting time (h/d)		
T1: < 7.8	235/286	1.00
T2: 7.8–10.4	227/277	0.91 (0.58–1.41)
T3: > 10.4	229/281	0.87 (0.56–1.35)
Ptrend		0.28
per 1 h/d	691/844	0.97 (0.91–1.03)
Total sedentary hours (h/d)		
T1: < 8.3	224/275	1.00
T2: 8.3–9.8	231/274	1.26 (0.80–1.98)
T3: > 9.8	220/274	0.98 (0.63–1.52)
Ptrend		0.75
per 1 h/d	675/823	0.98 (0.88–1.09)

Adjusted for: Age at diagnosis (years), cancer stage (I, II, III), cancer grade (I, II, III), study location (Calgary, Edmonton), breast cancer subtype (triple negative, HR + /HER2-, HR-/HER2 + , HR + /HER2 + ) and Charlson Comorbidity Index.

We observed higher lean mass percentage and lean fat mass ratio as well as lower BMI, waist circumference, WHR and fat mass percentage with ≥ 85% RDI (Table 2). Multivariable models showed that greater BMI, waist circumference, WHR and fat mass percentage were negatively associated with achieving ≥85% RDI while, lean mass percentage and lean fat mass ratio showed positive linear associations (Table 3). Compared with the lowest tertile of lean mass percentage and lean fat mass ratio, the highest tertile had twice the odds of ≥ 85% RDI (lean mass percentage: OR_T3 vs T1_=2.03, 95% CI = 1.28–3.22; lean fat mass ratio: OR_T3 vs T1_ = 2.06, 95% CI = 1.30–3.26). In contrast, BMI ≥ 30 kg/m^2^ compared with ≤ 25 kg/m^2^ (OR = 0.55, 95% CI = 0.35–0.86) or fat mass ≥ 46.4% compared with ≤ 40.1% (OR_T3 vs T1_ = 0.47, 95% CI = 0.30–0.76) approximately halved the odds of achieving ≥ 85% RDI (Table 3).

We observed null associations between RDI and most MVPA and SB exposures (Table 3). Only device-measured MVPA in bouts ≥10 min was associated with RDI (OR_T2 vs T1_ = 1.59, 95% CI = 1.02–2.48), though we found no linear trends. We found similar results when restricting the analysis to those who completed assessments prior to chemotherapy initiation (*N* = 615) (eTables 1–3).

In exploratory analyses, we observed associations between BMI (OR_per 1 kg/m2_ = 0.89, 95% CI = 0.80–0.99) and lean fat mass ratio (OR_per 0.1_ = 1.15, 95% CI = 1.00–1.32) with pCR (Table 4). We found a negative linear association between self-reported total PA and pCR (OR_per 10 METS-h/wk/yr_ = 0.90, 95% CI = 0.83–0.97). We had comparable results in a sensitivity analysis when we excluded 52 participants who received chemotherapy as part of a RCT (eTable 4).

**Table 4:** Logistic Regression for Health-Related Fitness, Body Composition, Physical Activity and Sedentary Behaviour with Pathologic Complete Response in the Alberta Moving Beyond Breast Cancer (AMBER) Study (*n* = 890).

	PCR/No. Participants	OR (95% CI)
**Aerobic Fitness**		
Relative VO_2_ peak (per 1 ml/kg/min)	27/98	1.08 (0.99–1.19)
Absolute VO_2_ peak (per 0.1 L/min)	27/98	1.00 (0.86–1.15)
**Muscular Strength**		
Upper body strength (per 10 kg)	33/106	0.69 (0.39–1.23)
Lower body strength (per 10 kg)	32/104	0.89 (0.77–1.04)
**Muscular Endurance**		
Upper body endurance (per 10 kg)	33/105	0.99 (0.96–1.01)
Lower body endurance (per 10 kg)	32/101	1.00 (0.99–1.00)
**Body Composition**		
Body mass index (per 1 kg/m^2^)	34/117	0.89 (0.80–0.99)
Waist circumference (per 1 cm)	34/117	0.96 (0.92–1.00)
Waistto-hip ratio (per 0.1)	34/117	0.72 (0.34–1.51)
Fat mass percentage (per 1%)	34/117	0.93 (0.86–1.00)
Lean body mass percentage (per 1%)	34/117	1.09 (0.99–1.18)
Lean-to-fat mass ratio (per 0.1)	34/117	1.15 (1.00–1.32)
**Physical Activity**		
Self-reported total physical activity (per 10 MET-h/wk/yr)	33/111	0.90 (0.83–0.97)
Self-reported recreational physical activity (per 10 MET-h/wk/yr)	33/111	0.87 (0.71–1.05)
Total energy expenditure (per MET-h/d)	33/109	1.00 (0.86–1.15)
Measured 10+ minutes bouts of MVPA (per 1 h/d)	33/109	1.69 (0.58–4.95)
Average daily steps (per 1000 steps/d)	33/108	1.04 (0.90–1.22)
**Sedentary Behaviours**		
Self-reported average sitting time (per 1 h/d)	33/110	0.88 (0.76–1.02)
Total sedentary hours (per 1 h/d)	33/108	1.15 (0.87–1.52)

Adjusted for: Age at diagnosis (years), cancer stage (I, II, III), cancer grade (I, II, III), study location (Calgary, Edmonton), breast cancer subtype (triple negative, HR + /HER2-, HR-/HER2 + , HR + /HER2 + ), Charlson Comorbidity Index and, RDI.

## Discussion

This cohort is the first and largest prospective study ever conducted among breast cancer patients that includes direct measures of PA, SB, and a wide range of HRF parameters in the examination of chemotherapy outcomes. Of the 890 participants included in this analysis, 726 (81.6%) completed curative-intent chemotherapy with ≥85% RDI. We observed that greater aerobic fitness and body strength were associated with a higher likelihood of completing chemotherapy. Consistent with this observation, measurements of greater relative lean mass were associated with more successful chemotherapy delivery whereas indicators of elevated levels of adiposity were associated with suboptimal RDI. In this cohort, we found mainly null associations between RDI and PA and SB. In exploratory analyses, greater lean-to-fat mass ratio and lower BMI were associated with better pCR. We also observed a linear relation between higher self-reported total PA and lower rates of pCR.

Pre-treatment physical fitness has been associated with a higher likelihood of achieving ≥85% RDI in exercise trials conducted among breast cancer patients [10, 11]. Specifically, An et al. [10], noted that breast cancer patients with the highest 20% compared with the lowest 80% of absolute VO_2peak_ were twice as likely to complete ≥ 85% RDI. Likewise, Groen et al. [11], observed that low pre-treatment physical fitness, were associated with 34% lower odds to complete ≥85% RDI. Consistent with past research, our participants with greater relative VO_2peak_ before chemotherapy treatment were more likely to achieve ≥85% RDI, supporting previous evidence on a favourable association between aerobic fitness and chemotherapy outcomes. These improved rates of chemotherapy completion and pCR seen among fitter breast cancer patients may occur through a combination of mitigating treatment-related side effects and biological adaptations to exercise, such as improving angiogenesis and blood perfusion and normalizing tumour vasculature [22, 23].

A systematic review on body composition and chemotherapy toxicities in breast cancer patients reported that most studies focused on assessments of sarcopenia via skeletal muscle index [12]. Overall significant associations between reduced skeletal muscle index with chemotherapy toxicities were observed, yet Lewis et al. highlighted that the diagnosis of sarcopenia based only on low skeletal muscle index does not consider muscle strength, an important aspect of the sarcopenia definition [12]. In another breast cancer study that examined muscular strength, patients with greater pre-treatment chest strength were nearly twice as likely to achieve ≥85% RDI [10]. One study in the review that considered fat and lean mass percentages obtained via DXA noted an increased (HR_per 5% fat mass_ = 1.21, 95%CI = 1.05–1.38) and decreased (HR_per 5% lean mass_ = 0.83, 95%CI = 0.72–0.96) risk of toxicity-induced modifications of treatment, respectively [12]. As with past reports, we found that fat mass percentage was negatively associated with successful chemotherapy completion while lean mass percentage and lean-to-fat mass ratio were positively associated. The linear associations between upper and lower absolute strength measures with ≥85% RDI are consistent with previous research. While we were unable to assess muscle quality directly, these results do suggest that relative lean mass and muscle function are key factors for breast cancer patients’ chemotherapy success. Notably, chemotherapy drugs are administered based on body surface area that is a product of height and weight but not body composition [24, 25]. Yet, in the general oncology setting and breast cancer specifically, the amount of lean soft tissues varies widely even for patients with the same body surface area [24, 25]. Given that drug metabolism occurs in lean soft tissues, a reduction in this tissue as seen in sarcopenia is thought to affect pharmacokinetics by reducing the volume of distribution, protein binding, metabolism, and clearance of drugs [24].

While past results support exercise during breast cancer treatment and have not shown that exercise would impede treatment tolerance, a specific exercise prescription to improve treatment outcomes has not yet been defined [9, 26]. The associations between PA and chemotherapy outcomes have largely centered around the period during active treatment [9]. Past trials have focused on breast cancer patients engaging in exercise at 55–85% of their maximal workloads [9]. We found no associations with past-year self-reported total or recreational PA and RDI. In the baseline accelerometry-assessed PA, ≥10 min bouts of MVPA (h/d), but not total energy expenditure or average daily step counts, were associated with better chemotherapy completion. This MVPA and RDI result corresponds most closely with previous exercise interventions (e.g., intensity, timing) conducted in breast cancer patients. These results suggest a relevant intensity and timeframe for exercise to be most impactful for chemotherapy completion [11]. This observation is supported by Groen et al. [11], who reported that MVPA program during treatment modified the relationship between pre-treatment fitness levels and chemotherapy tolerance.

To the best of our knowledge, this study is the first to examine SB and RDI directly, although we did not observe any associations with both self-reported and device-measured sedentary time. The accumulation of whole-body and visceral fat among cancer patients is more strongly associated with higher levels of objectively measured sedentary time than either PA or cardiorespiratory fitness [27]. These results highlight the potential importance of SB in the body composition and RDI relationship. Moreover, increased device-measured SB during adjuvant chemotherapy has been associated with worse symptoms such as fatigue, pain, physical and cognitive functioning [28]. Since patient symptoms may lead to dose reductions or delays in chemotherapy treatment, these symptoms could be a pathway between SB and RDI. We acknowledge that the pathway between SB, symptoms and RDI remains hypothetical currently and requires further investigation.

Previous studies have shown mixed associations between BMI-defined obesity and pCR after neoadjuvant breast cancer treatment [29–33], with few exploring other body composition measures, often reporting null associations in studies with small sample sizes [34, 35]. Similarly, pre-diagnosis or at-diagnosis total and recreational PA [29, 36, 37] have shown null associations with pCR. We observed associations between BMI, lean fat mass ratio, and self-reported total PA with pCR. While our findings support previously reported associations between obesity and pCR, the unexpected result for total PA estimate needs further investigation with consideration of domain- and intensity-specific PA. We acknowledge that the unexpected results for the association between pCR and total PA before diagnosis may be attributable to the large number of analyses without adjustment for multiple comparisons, the small sample size, and the reliance on self-report of PA for the year prior to diagnosis.

Our study strengths include: a large prospective sample; several direct measurements for HRF, body composition, PA, and SB exposures; and robust medical chart abstractions for RDI and pCR outcomes. The limitations were: the small sample size for the pCR analyses; the lack of data on reasons why participants had < 85% RDI; and the inability of some participants to complete the objective HRF measurements. While we mitigated the potential effects of a healthy sample bias by including the participants who could not complete the exercise tests because of medical reasons, participants could still be missing due to technical, logistical, and personal preferences. In addition, our results can only be generalized to similar predominantly White female breast cancer cases.

## Conclusion

Given the growing population of breast cancer patients and the widespread use of chemotherapy as part of standard treatment to improve prognostic outcomes in this group, identifying relevant factors for optimal delivery of chemotherapy while mitigating associated adverse events is paramount for maximizing curative outcomes. We found that 81.6% of AMBER participants achieved ≥85% RDI, and identified that greater relative aerobic fitness, muscular strength, and healthy body composition were relevant factors for better RDI. Future investigations should examine diverse measures of HRF and body composition as well as novel indicators such as SB since better methods for individualizing chemotherapy dosage and improving completion rates may result. Moreover, future studies of the longitudinal trajectories of these modifiable HRF, PA, and SB factors may elicit insights for treatment success and prognostic outcomes. Finally, future research on prehabilitation exercise interventions to improve HRF prior to adjuvant chemotherapy is needed given the potential benefits for treatment tolerance and response. These results support further consideration of incorporating structured exercise programs into treatment pathways to mitigate adverse effects and improve treatment tolerance.

## Supplementary information

Supplementary Tables

## Data Availability

The data underlying this article will be shared on reasonable request made to the corresponding author.
