# Supplementary material for: Associations of health-related fitness and physical activity with chemotherapy outcomes in breast cancer
Source: Br J Cancer. 2026 Mar 27;134(10):1459–67. doi: 10.1038/s41416-026-03384-3 (PMC13133392; doi:10.1038/s41416-026-03384-3)
Supplement: Supplementary file 1 — Supplementary Tables [file 41416_2026_3384_MOESM1_ESM.docx]

Supplementary Tables: Associations of Health-Related Fitness and Physical Activity with Chemotherapy Outcomes in Breast Cancer

Supplementary Table 1: Health Related Fitness, Body Composition, Physical Activity and Sedentary Behaviour Characteristics of Participants by Relative Dose Intensity Status in the Alberta Moving Beyond Breast Cancer (AMBER) study (n=615).

| **Exposures** | **All**  **Median (IQR)** | **RDI <0.85%**  **Median (IQR)** | **RDI ≥0.85%**  **Median (IQR)** | **P-value** |
| --- | --- | --- | --- | --- |
| Participants | 615 | 111 | 504 |  |
| **Aerobic fitness** |  |  |  |  |
| Relative VO_2_ peak (ml/kg/min) | 26.8 (23.0-31.1) | 24.7 (21.8-29.0) | 27.1 (23.3-31.3) | <0.01 |
| Absolute VO_2_ peak (L/min) | 1.9 (1.7-2.2) | 1.9 (1.6-2.1) | 1.9 (1.7-2.2) | 0.04 |
| **Muscular strength** |  |  |  |  |
| Upper body strength (kg) | 35.7 (29.7-41.6) | 33.0 (27.0-40.1) | 35.7 (29.7-42.0) | <0.01 |
| Lower body strength (kg) | 95.0 (77.0-118.9) | 89.1 (71.3-107.0) | 95.1 (77.3-118.9) | 0.01 |
| **Muscular endurance** |  |  |  |  |
| Upper body endurance (kg) | 455.0 (341.0-590.9) | 426.5 (325.0-545.5) | 475.0 (341.0-600.0) | 0.07 |
| Lower body endurance (kg) | 1159.1 (776.0-1649.0) | 1150.0 (750.0-1500.0) | 1159.1 (777.3-1659.1) | 0.51 |
| **Body composition** |  |  |  |  |
| Body mass index (kg/m^2^) | 26.2 (23.1-30.3) | 27.3 (24.2-31.1) | 26.1 (23.0-30.0) | 0.02 |
| Waist circumference (cm) | 91.2 (82.2-101.0) | 94.0 (83.5-105.0) | 90.2 (82.0-100.1) | 0.01 |
| Waist-to-hip ratio | 0.9 (0.8-0.9) | 0.9 (0.8-0.9) | 0.9 (0.8-0.9) | 0.07 |
| Fat mass percentage (%) | 43.3 (37.8-47.9) | 45.0 (40.2-49.5) | 42.9 (37.2-47.6) | <0.01 |
| Lean mass percentage (%) | 53.5 (49.1-58.5) | 52.1 (47.8-56.2) | 53.9 (49.5-59.2) | <0.01 |
| Lean-to-fat mass ratio | 1.2 (1.0-1.5) | 1.2 (1.0-1.4) | 1.3 (1.0-1.6) | <0.01 |
| **Physical Activity** |  |  |  |  |
| Self-reported total physical activity (MET-h/wk/yr) | 122.1 (85.1-172.8) | 122.5 (85.5-164.4) | 121.6 (85.1-175.5) | 0.52 |
| Self-reported recreational physical activity (MET-h/wk/yr) | 20.2 (9.0-39.0) | 19.0 (8.6-39.7) | 20.8 (9.0-38.9) | 0.80 |
| Total energy expenditure (MET-h/d) | 17.0 (15.2-19.3) | 16.5 (15.1-19.1) | 17.2 (15.3-19.4) | 0.14 |
| Measured 10+ minutes bouts of MVPA (h/d) | 0.2 (0.1-0.5) | 0.2 (0.0-0.5) | 0.2 (0.1-0.5) | 0.82 |
| Average daily steps | 6807.1 (5101.7-8965.7) | 5988.9 (4684.0-9051.0) | 6900.6 (5198.0-8965.5) | 0.22 |
| **Sedentary Behaviours** |  |  |  |  |
| Self-reported average sitting time (h/d) | 9.0 (6.9-11.0) | 9.5 (7.5-11.3) | 9.0 (6.8-11.0) | 0.39 |
| Total sedentary hours (h/d) | 9.1 (8.0-10.2) | 9.2 (7.9-10.3) | 9.1 (8.0-10.2) | 0.68 |

MVPA=moderate-to-vigorous physical activity; P-values based on the Wilcoxon rank-sum

Supplementary Table 2. Logistic Regression for Health-Related Fitness, Body Composition, Physical Activity and Sedentary Behaviour with ≥85% Relative Dose Intensity in the Alberta Moving Beyond Breast Cancer (AMBER) study (n=615).

|  | Events ≥85%/ No. Participants | OR (95% CI) |
| --- | --- | --- |
| **Aerobic fitness** |  |  |
| Relative VO_2_peak (ml/kg/min) |  |  |
| Did not complete assessment | 33/45 | 0.84 (0.38-1.84) |
| T1: <24.2 | 127/166 | 1.00 |
| T2: 24.2-28.9 | 133/156 | 1.75 (0.98-3.13) |
| T3: >28.9 | 152/173 | 1.86 (1.01-3.42) |
| Ptrend |  | 0.047 |
| per 1 ml/kg/min | 455/540 | 1.05 (1.00-1.10) |
| Absolute VO_2_ peak (L/min) |  |  |
| Did not complete assessment | 33/45 | 0.65 (0.29-1.44) |
| T1: <1.76 | 130/161 | 1.00 |
| T2: 1.76-2.06 | 137/169 | 0.93 (0.53-1.65) |
| T3: >2.06 | 145/165 | 1.50 (0.77-2.91) |
| Ptrend |  | 0.212 |
| per 0.1 L/min | 455/540 | 1.05 (0.97-1.13) |
| **Muscular Strength** |  |  |
| Upper body strength (kg) |  |  |
| Did not complete assessment | 60/76 | 1.28 (0.64-2.54) |
| T1: <32.1 | 138/180 | 1.00 |
| T2: 32.1-40.1 | 152/177 | 1.63 (0.93-2.86) |
| T3: >40.1 | 136/156 | 1.61 (0.86-2.98) |
| Ptrend |  | 0.113 |
| per 10 kg | 486/589 | 1.24 (0.95-1.61) |
| Lower body strength (kg) |  |  |
| Did not complete assessment | 24/35 | 0.68 (0.30-1.57) |
| T1: <83.3 | 160/203 | 1.00 |
| T2: 83.3-107.0 | 141/165 | 1.28 (0.73-2.26) |
| T3: >107.0 | 155/178 | 1.55 (0.88-2.76) |
| Ptrend |  | 0.046 |
| per 10 kg | 480/581 | 1.08 (1.00-1.17) |
| **Muscular Endurance** |  |  |
| Upper body endurance (total kg) |  |  |
| Did not complete assessment | 60/76 | 0.96 (0.47-1.95) |
| T1: <384 | 141/172 | 1.00 |
| T2: 384-552.3 | 132/168 | 0.74 (0.43-1.29) |
| T3: >552.3 | 149/168 | 1.47 (0.78-2.77) |
| Ptrend |  | 0.269 |
| per 10 kg | 482/584 | 1.01 (0.99-1.02) |
| Lower body endurance (total kg) |  |  |
| Did not complete assessment | 24/35 | 0.55 (0.23-1.31) |
| T1:<922.7 | 153/181 | 1.00 |
| T2: 922.7-1475.0 | 146/179 | 0.83 (0.47-1.46) |
| T3: >1475.0 | 147/175 | 1.00 (0.55-1.80) |
| Ptrend |  | 0.281 |
| per 10 kg | 470/570 | 1.00 (1.00-1.00) |
| **Body Composition** |  |  |
| Body mass index (kg/m^2^) |  |  |
| ≤24.9 | 212/248 | 1.00 |
| 25.0-29.9 | 166/203 | 0.79 (0.47-1.33) |
| ≥30 | 126/164 | 0.63 (0.37-1.08) |
| Ptrend |  | 0.028 |
| per 1 kg/m^2^ | 504/615 | 0.96 (0.93-1.00) |
| Waist circumference (cm) |  |  |
| <88.0 | 226/265 | 1.00 |
| ≥88.0 | 278/350 | 0.72 (0.46-1.12) |
| Ptrend |  | 0.013 |
| per 1 cm | 504/615 | 0.98 (0.97-1.00) |
| Waist-to-hip ratio |  |  |
| ≤0.84 | 186/220 | 1.00 |
| ≥0.85 | 318/395 | 0.83 (0.52-1.32) |
| Ptrend |  | 0.118 |
| per 0.1 | 504/615 | 0.77 (0.55-1.07) |
| Fat mass percentage (%) |  |  |
| T1: <40.1 | 180/220 | 1.00 |
| T2: 40.1-46.4 | 164/201 | 0.71 (0.40-1.25) |
| T3: >46.4 | 148/193 | 0.53 (0.30-0.93) |
| Ptrend |  | 0.029 |
| per 1 % | 492/599 | 0.96 (0.93-1.00) |
| Lean body mass percentage (%) |  |  |
| T1: <50.6 | 151/197 | 1.00 |
| T2: 50.6-56.3 | 162/197 | 1.39 (0.84-2.31) |
| T3: >56.3 | 179/205 | 1.78 (1.02-3.08) |
| Ptrend |  | 0.027 |
| per 1 % | 492/599 | 1.04 (1.00-1.08) |
| Lean-to-fat mass ratio |  |  |
| T1: <50.2 | 148/194 | 1.00 |
| T2: 50.2-56.5 | 165/200 | 1.45 (0.88-2.42) |
| T3: >56.5 | 179/205 | 1.82 (1.04-3.16) |
| Ptrend |  | 0.028 |
| per 0.1 | 492/599 | 1.07 (1.0-1.13) |
| **Physical Activity** |  |  |
| Self-reported total non-sedentary physical activity (MET-h/wk/yr) |  |  |
| T1: <98.1 | 159/199 | 1.00 |
| T2: 98.1-152.7 | 154/186 | 1.14 (0.67-1.94) |
| T3: >152.7 | 168/201 | 1.09 (0.63-1.87) |
| Ptrend |  | 0.77 |
| per 10 MET-h/wk/yr | 481/586 | 0.99 (0.96-1.03) |
| Self-reported recreational physical activity (MET-h/wk/yr) |  |  |
| T1: <12.7 | 166/201 | 1.00 |
| T2: 12.7-31.5 | 149/183 | 0.91 (0.53-1.55) |
| T3: >31.5 | 166/202 | 0.91 (0.54-1.55) |
| Ptrend |  | 0.80 |
| per 10 MET-h/wk/yr | 481/586 | 0.99 (0.92-1.07) |
| Total energy expenditure (MET-h/d) |  |  |
| T1: <15.8 | 149/187 | 1.00 |
| T2: 15.8-18.2 | 150/184 | 1.03 (0.60-1.75) |
| T3 >18.2 | 179/213 | 1.08 (0.63-1.84) |
| Ptrend |  | 0.62 |
| per 1 MET-h/d | 478/584 | 1.02 (0.95-1.10) |
| Measured 10+ minutes bouts of MVPA (h/d) |  |  |
| T1: <0.1 | 147/183 | 1.00 |
| T2: 0.1-0.3 | 165/199 | 1.19 (0.69-2.04) |
| T3: >0.3 | 166/202 | 1.05 (0.61-1.81) |
| Ptrend |  | 0.98 |
| per 1 h/d | 478/584 | 0.99 (0.49-1.98) |
| Average daily steps (steps/d) |  |  |
| T1: <5506.0 | 152/191 | 1.00 |
| T2: 5506.0-8061.5 | 155/185 | 1.12 (0.65-1.94) |
| T3: >8061.5 | 169/201 | 1.15 (0.70-1.98) |
| Ptrend |  | 0.78 |
| per 1000 steps/d | 476/577 | 1.01 (0.94-1.09) |
| **Sedentary Behaviours** |  |  |
| Self-reported average sitting time (h/d) |  |  |
| T1: <7.8 | 173/206 | 1.00 |
| T2: 7.8-10.4 | 151/190 | 0.73 (0.43-1.23) |
| T3: >10.4 | 157/189 | 0.90 (0.52-1.55) |
| Ptrend |  | 0.44 |
| per 1 h/d | 481/585 | 0.97 (0.90-1.04) |
| Total sedentary hours (h/d) |  |  |
| T1: <8.3 | 149/182 | 1.00 |
| T2: 8.3-9.8 | 167/198 | 1.29 (0.74-2.24) |
| T3: >9.8 | 160/197 | 1.09 (0.63-1.86) |
| Ptrend |  | 0.96 |
| per 1 h/d | 476/577 | 1.00 (0.87-1.14) |

Adjusted for: Age at diagnosis (years), cancer stage (I, II, III), cancer grade (I, II, III), study location (Calgary, Edmonton), breast cancer subtype (triple negative, HR+/HER2-, HR-/HER2+, HR+/HER2+) and Charlson Comorbidity Index

Supplementary Table 3. Logistic Regression for Health-Related Fitness, Body Composition, Physical Activity and Sedentary Behaviour with Pathologic Complete Response in the Alberta Moving Beyond Breast Cancer (AMBER) study (n=615).

|  | PCR/  No. Participants | OR (95% CI) |
| --- | --- | --- |
| **Aerobic Fitness** |  |  |
| Relative VO_2_ peak (per 1 ml/kg/min) | 9/27 | 1.03 (0.76-1.39) |
| Absolute VO_2_ peak (per 0.1 L/min) | 9/27 | 0.75 (0.41-1.39) |
| **Muscular Strength** |  |  |
| Upper body strength (per 10 kg) | 12/28 | 1.15 (0.29-4.61) |
| Lower body strength (per 10 kg) | 11/28 | 0.79 (0.50-1.24) |
| **Muscular Endurance** |  |  |
| Upper body endurance (per 10 kg) | 11/28 | 0.79 (0.50-1.24) |
| Lower body endurance (per 10 kg) | 11/28 | 1.00 (0.99-1.01) |
| **Body Composition** |  |  |
| Body mass index (per 1 kg/m^2^) | 12/32 | 0.62 (0.35-1.10) |
| Waist circumference (per 1 cm) | 12/32 | 0.91 (0.80-1.04) |
| Waist-to-hip ratio (per 0.1) | 12/32 | 0.37 (0.09-1.50) |
| Fat mass percentage (per 1 %) | 12/32 | 0.94 (0.80-1.10) |
| Lean body mass percentage (per 1 %) | 12/32 | 1.07 (0.91-1.27) |
| Lean-to-fat mass ratio (per 0.1) | 12/32 | 1.09 (0.84-1.43) |
| **Physical Activity** |  |  |
| Self-reported total physical activity (per 10 MET-h/wk/yr) | 12/31 | 0.90 (0.77-1.05) |
| Self-reported recreational physical activity  (per 10 MET-h/wk/yr) | 12/31 | 0.77 (0.52-1.15) |
| Total energy expenditure (per MET-h/d) | 12/29 | 1.04 (0.79-1.36) |
| Measured 10+ minutes bouts of MVPA (per 1 h/d) | 12/29 | 0.98 (0.02-42.2) |
| Average daily steps (per 1000 steps/d) | 11/28 | 1.05 (0.79-1.40) |
| **Sedentary Behaviours** |  |  |
| Self-reported average sitting time (per 1 h/d) | 12/30 | 0.81 (0.60-1.09) |
| Total sedentary hours (per 1 h/d) | 11/28 | 1.18 (0.76-1.84) |

Adjusted for: Age at diagnosis (years), cancer stage (I, II, III), cancer grade (I, II, III), study location (Calgary, Edmonton), breast cancer subtype (triple negative, HR+/HER2-, HR-/HER2+, HR+/HER2+), Charlson Comorbidity Index and, RDI

Supplementary Table 4. Sensitivity Analysis for Health-Related Fitness, Body Composition, Physical Activity and Sedentary Behaviour with ≥85% RDI among Participants without RCT Chemotherapy (n=839).

|  | Events ≥85%/  No. Participants | OR (95% CI) |
| --- | --- | --- |
| **Aerobic fitness** |  |  |
| Relative VO_2_ peak (ml/kg/min) |  |  |
| Did not complete assessment | 47/60 | 1.29 (063-2.64) |
| T1: <24.2 | 167/222 | 1.00 |
| T2: 24.2-28.9 | 189/222 | 1.79 (1.09-2.92) |
| T3: >28.9 | 195/220 | 2.12 (1.23-3.64) |
| Ptrend |  | 0.004 |
| per 1 ml/kg/min | 551/664 | 1.06 (1.02-1.11) |
| Absolute VO_2_ peak (L/min) |  |  |
| Did not complete assessment | 47/60 | 0.91 (0.44-1.90) |
| T1: <1.76 | 185/227 | 1.00 |
| T2: 1.76-2.06 | 179/223 | 0.82 (0.51-1.34) |
| T3: >2.06 | 187/214 | 1.32 (0.74-2.33) |
| Ptrend |  | 0.26 |
| per 0.1 L/min | 551/664 | 1.04 (0.97-1.11) |
| **Muscular Strength** |  |  |
| Upper body strength (kg) |  |  |
| Did not complete assessment | 73/91 | 1.44 (0.77-2.70) |
| T1: <32.1 | 186/242 | 1.00 |
| T2: 32.1-40.1 | 208/244 | 1.55 (0.96-2.50) |
| T3: >40.1 | 195/226 | 1.45 (0.87-2.43) |
| Ptrend |  | 0.07 |
| per 10 kg | 589/712 | 1.22 (0.99-1.53) |
| Lower body strength (kg) |  |  |
| Did not complete assessment | 38/49 | 1.21 (0.56-2.63) |
| T1: <83.3 | 218/275 | 1.00 |
| T2: 83.3-107.0 | 194/228 | 1.25 (0.77-2.02) |
| T3: >107.0 | 206/242 | 1.29 (0.80-2.09) |
| Ptrend |  | 0.08 |
| per 10 kg | 618/745 | 1.06 (0.99-1.13) |
| **Muscular Endurance** |  |  |
| Upper body endurance (total kg) |  |  |
| Did not complete assessment | 74/92 | 1.12 (0.59-2.12) |
| T1: <384 | 197/240 | 1.00 |
| T2: 384-552.3 | 181/234 | 0.70 (0.44-1.12) |
| T3: >552.3 | 206/232 | 1.50 (0.87-2.57) |
| Ptrend |  | 0.15 |
| per 10 kg | 584/706 | 1.01 (0.99-1.02) |
| Lower body endurance (total kg) |  |  |
| Did not complete assessment | 38/49 | 0.98 (0.44-2.17) |
| T1:<922.7 | 212/252 | 1.00 |
| T2: 922.7-1475.0 | 192/239 | 0.75 (0.46-1.20) |
| T3: >1475.0 | 200/238 | 0.98 (0.59-1.63) |
| Ptrend |  | 0.65 |
| per 10 kg | 605/730 | 1.00 (0.99-1.00) |
| **Body Composition** |  |  |
| Body mass index (kg/m^2^) |  |  |
| ≤24.9 | 293/335 | 1.00 |
| 25.0-29.9 | 234/287 | 0.69 (0.44-1.08) |
| ≥30 | 164/217 | 0.52 (0.32-0.82) |
| Ptrend |  | 0.005 |
| per 1 kg/m^2^ | 690/839 | 0.96 (0.93-0.99) |
| Waist circumference (cm) |  |  |
| <88.0 | 307/356 | 1.00 |
| ≥88.0 | 383/483 | 0.68 (0.46-1.00) |
| Ptrend |  | 0.003 |
| per 1 cm | 690/839 | 0.98 (0.97-0.99) |
| Waist-to-hip ratio |  |  |
| ≤0.84 | 265/308 | 1.00 |
| ≥0.85 | 425/531 | 0.70 (0.47-1.06) |
| Ptrend |  | 0.022 |
| per 0.1 | 690/839 | 0.72 (0.54-0.95) |
| Fat mass percentage (%) |  |  |
| T1: <40.1 | 245/276 | 1.00 |
| T2: 40.1-46.4 | 227/275 | 0.68 (0.41-1.11) |
| T3: >46.4 | 205/259 | 0.49 (0.30-0.79) |
| Ptrend |  | 0.002 |
| per 1 % | 677/820 | 0.96 (0.93-0.98) |
| Lean body mass percentage (%) |  |  |
| T1: <50.6 | 205/270 | 1.00 |
| T2: 50.6-56.3 | 228/274 | 1.46 (0.95-2.26) |
| T3: >56.3 | 244/276 | 1.98 (1.22-3.20) |
| Ptrend |  | 0.002 |
| per 1 % | 677/820 | 1.05 (1.02-1.08) |
| Lean-to-fat mass ratio |  |  |
| T1: <50.2 | 205/270 | 1.00 |
| T2: 50.2-56.5 | 228/274 | 1.48 (0.96-2.28) |
| T3: >56.5 | 244/276 | 2.00 (1.24-3.25) |
| Ptrend |  | 0.003 |
| per 0.1 | 677/820 | 1.08 (1.03-1.14) |
| **Physical Activity** |  |  |
| Self-reported total physical activity (MET-h/wk/yr) |  |  |
| T1: <98.1 | 221/274 | 1.00 |
| T2: 98.1-152.7 | 212/260 | 1.06 (0.68-1.65) |
| T3: >152.7 | 225/264 | 1.26 (0.78-2.03) |
| Ptrend |  | 0.95 |
| per 10 MET-h/wk/yr |  | 1.00 (0.97-1.03) |
| Self-reported recreational physical activity (MET-h/wk/yr) |  |  |
| T1: <12.7 | 223/270 | 1.00 |
| T2: 12.7-31.5 | 216/263 | 0.98 (0.62-1.54) |
| T3: >31.5 | 219/265 | 0.99 (0.63-1.56) |
| Ptrend |  | 0.66 |
| per 10 MET-h/wk/yr |  | 0.98 (0.92-1.05) |
| Total energy expenditure (MET-h/d) |  |  |
| T1: <15.8 | 204/259 | 1.00 |
| T2: 15.8-18.2 | 224/263 | 1.48 (0.93-2.35) |
| T3 >18.2 | 220/266 | 1.12 (0.71-1.76) |
| Ptrend |  | 0.48 |
| per 1 MET-h/day |  | 1.02 (0.96-1.09) |
| Measured 10+ minutes bouts of MVPA (h/d) |  |  |
| T1: <0.1 | 207/261 | 1.00 |
| T2: 0.1-0.3 | 223/263 | 1.51 (0.95-2.41) |
| T3: >0.3 | 218/264 | 1.21 (0.77-1.91) |
| Ptrend |  | 0.970 |
| per 1 h/d |  | 0.99 (0.57-1.73) |
| Average daily steps (steps/d) |  |  |
| T1: <5506.0 | 202/255 | 1.00 |
| T2: 5506.0-8061.5 | 219/260 | 1.18 (0.74-1.88) |
| T3: >8061.5 | 223/262 | 1.30 (0.81-2.09) |
| Ptrend |  | 0.21 |
| per 1000 steps/d | 644/777 | 1.04 (0.97-1.12) |
| **Sedentary Behaviours** |  |  |
| Self-reported average sitting time (h/d) |  |  |
| T1: <7.8 | 225/270 | 1.00 |
| T2: 7.8-10.4 | 214/261 | 0.83 (0.52-1.31) |
| T3: >10.4 | 218/264 | 0.87 (0.55-1.39) |
| Ptrend |  | 0.252 |
| per 1 h/d |  | 0.97 (0.91-1.03) |
| Total sedentary hours (h/d) |  |  |
| T1: <8.3 | 210/256 | 1.00 |
| T2: 8.3-9.8 | 224/263 | 1.32 (0.82-2.13) |
| T3: >9.8 | 210/258 | 1.03 (0.65-1.63) |
| Ptrend |  | 0.80 |
| per 1 h/d |  | 0.99 (0.88-1.11) |

Adjusted for: Age at diagnosis (years), cancer stage (I, II, III), cancer grade (I, II, III), study location (Calgary, Edmonton), breast cancer subtype (triple negative, HR+/HER2-, HR-/HER2+, HR+/HER2+), and Charlson Comorbidity Index.
